# Laboratory variation in the grading of dysplasia of duodenal adenomas in familial adenomatous polyposis patients

**DOI:** 10.1007/s10689-022-00320-1

**Published:** 2022-11-19

**Authors:** E. Soons, P. D. Siersema, L. M. A. van Lierop, T. M. Bisseling, M. C. A. van Kouwen, I. D. Nagtegaal, R. S. van der Post, F. Atsma

**Affiliations:** 1 Department of Gastroenterology and Hepatology, Radboud Institute for Health Sciences, Radboud University Medical Center, Nijmegen, The Netherlands; 2 Department of Pathology, Radboud Institute for Molecular Life Sciences, Radboud University Medical Center, Nijmegen, The Netherlands; 3 Department of IQ Healthcare, Radboud Institute for Health Sciences, Radboud University Medical Center, Nijmegen, The Netherlands

**Keywords:** Pathology, Gastroenterology, Familial adenomatous polyposis (FAP), Duodenal polyposis

## Abstract

To prevent duodenal and ampullary cancer in familial adenomatous polyposis (FAP) patients, a diagnosis of high grade dysplasia (HGD) plays an important role in the clinical management. Previous research showed that FAP patients are both over- and undertreated after a misdiagnosis of HGD, indicating unwarranted variation. We aimed to investigate the laboratory variation in dysplasia grading of duodenal adenomas and explore possible explanations for this variation. We included data from all Dutch pathology laboratories between 1991 and 2020 by retrieving histology reports from upper endoscopy specimens of FAP patients from the Dutch nationwide pathology databank (PALGA). Laboratory variation was investigated by comparing standardized proportions of HGD. To describe the degree of variation between the laboratories a factor score was calculated. A funnel plot was used to identify outliers. A total of 3050 specimens from 25 laboratories were included in the final analyses. The mean observed HGD proportion was 9.4%. The top three HGD-diagnosing laboratories diagnosed HGD 3.9 times more often than the lowest three laboratories, even after correcting for case-mix. No outliers were identified. Moderate laboratory variation was found in HGD diagnoses of duodenal tissue of FAP patients after adjusting for case-mix. Despite the fact that no outliers were observed, there may well be room for quality improvement. Concentration of these patients in expertise centers may decrease variation. To further reduce unwarranted variation, we recommend (inter)national guidelines to become more uniform in their recommendations regarding duodenal tissue sampling and consequences of HGD diagnoses.

**Supplementary Information:**

The online version contains supplementary material available at 10.1007/s10689-022-00320-1.

## Introduction

Familial adenomatous polyposis (FAP) affects one in 10,000 individuals, which makes it the second most common inherited colorectal cancer (CRC) syndrome [1, 2]. A mutation in the adenomatous polyposis coli (*APC*) gene leads to the formation of ≥ 100 synchronous polyps distributed throughout the gastrointestinal tract [3, 4]. The standardization of colonic screening at a young age and subsequent preventive surgery has largely reduced mortality from CRC [5]. In addition, individuals with FAP have an increased risk of duodenal and ampullary cancer, with relative risks of 331 and 124, respectively, compared to the general population. Consequently, duodenal and ampullary cancer are nowadays the most common cause of cancer-related death in FAP [6, 7].

The histological grade of dysplasia in duodenal adenomas plays an important role in the decision-making process to prevent duodenal and/or ampullary cancer in two ways. First, international guidelines recommend starting duodenal surveillance at the age of 25–35 years [8–11]. The surveillance interval is traditionally determined by the Spigelman classification, which includes histological grading as one of four decisive parameters [12]. A diagnosis of high grade dysplasia (HGD) will lead to a shortened surveillance interval in many cases. Secondly, the presence of HGD is a relative indication for an endoscopic or surgical intervention as it is considered a risk factor for developing duodenal cancer [9, 11, 13–15].

As a result of the need to screen for dysplasia in duodenal tissue in FAP patients, duodenal tissue is routinely seen by pathologists. Yet, there are indications that misdiagnoses of dysplasia occur, which may have major clinical consequences. For instance, Sourouille et al. described the histopathological diagnoses of 52 duodenal specimens collected after radical surgical treatment [14]. Surgery was performed in FAP patients with endoscopically untreatable duodenal polyposis and/or an ampullary adenoma or Spigelman score IV with HGD at 2 successive endoscopic assessments three months apart and confirmed by two independent pathologists. They found that in eleven patients (21.2%) surgery was performed too late (i.e., cancer was already present), while in fourteen patients (29.7%) surgery was performed too early (i.e., no HGD or cancer was present). Both scenarios indicate inappropriate care, as the misdiagnosis of HGD led to both over- and undertreatment of duodenal polyposis.

Measuring institutional variation in clinical care may help identifying inappropriate care and/or suboptimal quality of care and may provide target points for quality improvement [16]. Variation in clinical care is an issue, especially if it is unwarranted. The latter may occur in clinical care when patients undergo care which is not indicated, as illustrated in the above-mentioned study by Sourouille et al. [17]. So far, no studies on laboratory variation in the grading of dysplasia in duodenal tissue have been published. In clinical practice, the guideline that is used for grading duodenal dysplasia is the same as for colorectal dysplasia [18]. In the latter, considerable interlaboratory variation has been reported in grading of colonic dysplasia, with 35% of laboratories reporting a significantly lower or higher frequency of HGD than average; however this information is not yet known for duodenal dysplasia grading [19]. If the same is true for duodenal tissue, there is need to reduce this variation to prevent over- and undertreatment of duodenal polyposis.

The aim of the current study was to investigate the extent of laboratory variation in the histological dysplasia grading of duodenal adenomas from patients with FAP in a nationwide cohort and, if present, to identify possible explanations for this variation.

## Methods

### Data extraction

All data were extracted from PALGA, the Dutch nationwide pathology databank. PALGA contains excerpts of all pathology reports from Dutch pathology laboratories, with nationwide coverage since 1991 [20]. All PALGA data are pseudonymized by a trusted third party, securing that in the PALGA database no personally identifiable data are collected. Data from patients who refuse their data to be used for scientific research are excluded from the PALGA database. The scientific and privacy committee of PALGA approved the protocol of this study (Reference Number: 2020-41). Non-identifiable data makes this study to be exempted from ethical approval.

We identified all reports with one or more diagnoses of duodenal adenoma between 1991 and 2020 from patients diagnosed with FAP or who had a prior (sub)total colectomy, assuming they were also FAP-patients. If there were multiple records per patients, all reports were included.

### Laboratory, patient and specimen selection

**Fig. 1:**
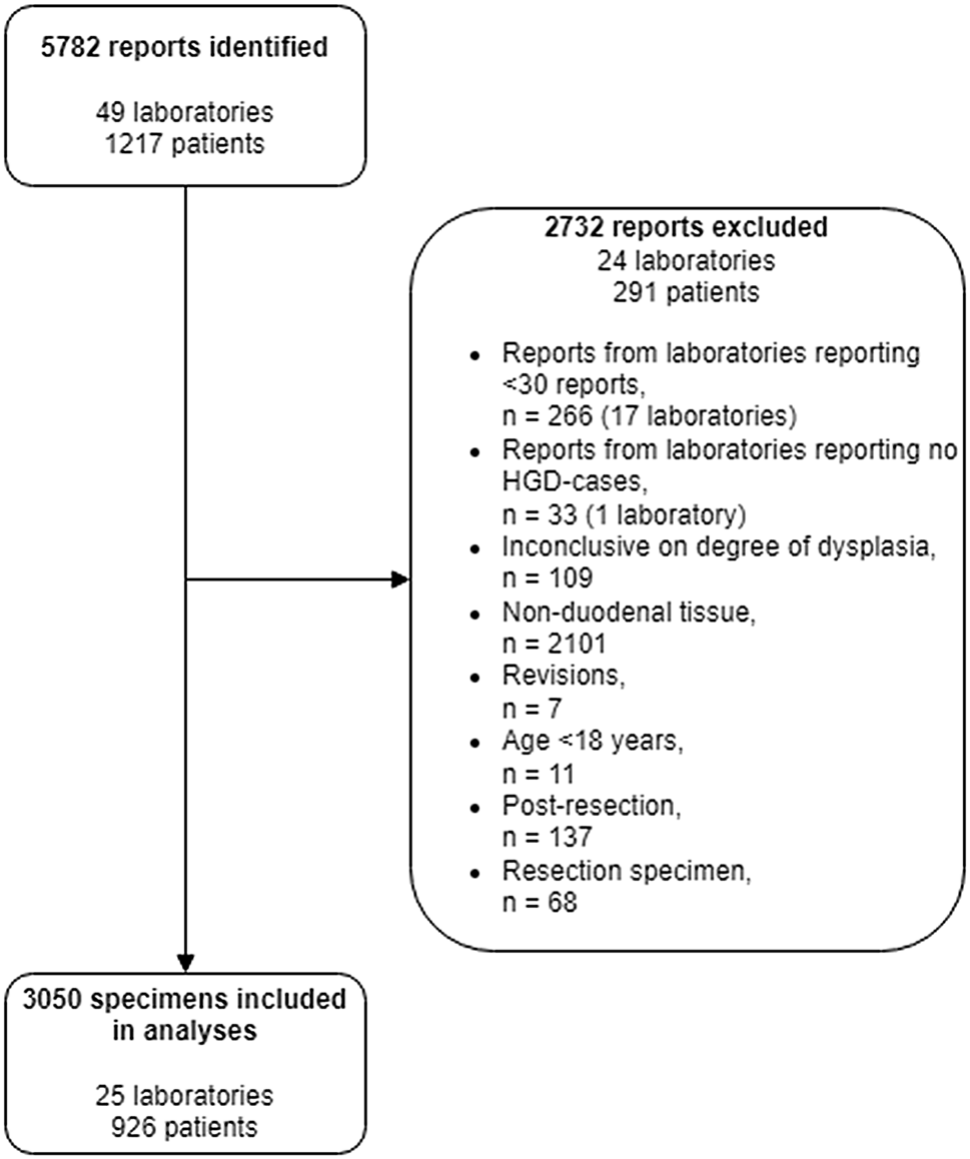
Flowchart representing reasons for exclusion for excluded reports

Based on the search criteria described above, a total of 5782 reports from 1217 patients and 49 laboratories were identified. To account for small sample variations, reports from laboratories with < 30 reports (n = 266) or without an HGD diagnosis (n = 33) over the total inclusion period were excluded from further analysis (see Fig. 1). To aim for uniformity in our dataset, pathology reports were also excluded if they were inconclusive on the degree of dysplasia (n = 109) or origin of the tissue (n = 2101), revised cases (n = 7), from patients < 18 years of age (n = 11) or from patients who previously underwent duodenal resection (n = 137). Furthermore, reports of resection specimens were excluded (n = 68) when information on the number, size and location of the duodenal specimens remained unclear in these cases. To correct for multiple paired measurements, we included one specimen per report. This was either the (first) specimen diagnosed with HGD, or, in absence of a HGD diagnosis, the first specimen that was described in the report.

The final dataset used for further analyses therefore consisted of 3050 specimens from 926 patients in 25 laboratories.

### Data collection

For each laboratory, we registered the type of laboratory (academic or general), number of patients and number of specimens. For each patient we extracted age, sex and total number of specimens. Only the first report per patient was used to describe these characteristics since multiple reports per patient were included for most patients (55.5%). For each specimen extracted, the year of the histology report, total number of removed specimens per report, degree of dysplasia (no dysplasia, LGD, HGD or carcinoma), morphological type (tubular, tubulovillous or villous), localization in the duodenum from first to fourth part (D1-D4), tissue from the duodenal papilla or not and tissue obtained by biopsy or not, was recorded.

Clinical characteristics were extracted manually. To validate the extracted data, 10% of the reports were double checked by three additional investigators (ES, MvK and TB). Multiple imputation was performed for the variable morphology, which had 26.3% missing data. All other variables were complete.

### Statistical analysis

#### Descriptive analysis

Overall laboratory characteristics were described with respect to laboratory type and number of patients and specimens. Overall patient characteristics were described with respect to age, sex and number of specimens. Specimen characteristics were described with respect to year of histology report, total number of removed specimens per report, location in duodenum, degree of dysplasia, morphology type and method used to obtain the tissue. Categorical variables are presented as frequencies and percentages. Continuous variables are reported as means ± standard deviation (SD) or medians (interquartile range (IQR)), in case of a skewed distribution.

#### Laboratory variation in proportion HGD diagnosis

Laboratories were compared by indirect standardization of HGD proportions. First, the observed proportion of HGD diagnoses per laboratory was assessed. Second, expected proportions of HGD diagnoses per laboratory were calculated with a multilevel logistic regression model. This model accounts for age, year of the report, number of specimens per report, localization in the duodenum, localization at the papilla major, morphology and method used to obtain the tissue (i.e., polypectomy or other). Localization in the duodenum at the papilla major, or the method of obtaining the tissue were significant predictors in univariate analysis. Nevertheless, as previous studies showed the significance of these variables, they were included in the final model regardless [14, 19]. See Supplementary Table 1 for the contribution of each variable to the final model. Third, the observed HGD proportions were divided by the expected (adjusted) HGD proportions per laboratory. This led to an observed/expected-ratio (O/E-ratio) indicating less HGD diagnoses than expected when < 1, or more HGD diagnoses than expected when > 1. Fourth, by multiplying the O/E-ratio with the overall HGD proportion, the standardized HGD proportion was calculated.

To quantify the amount of interlaboratory variation in HGD diagnoses, a factor score was calculated. Factor scores are used to illustrate by which factor the highest score differs from the lowest score. The factor score was calculated by dividing the mean proportion of the highest three laboratories by the mean proportion of the lowest 3 laboratories. A variation of factor two is considered to be modest [21].

#### Funnel plots

Funnel plots were constructed to detect outliers. In short, these are frequently used control charts in which an outcome measure for a unit of analysis (e.g., a histopathologic laboratory) is plotted against a measure for the laboratory size (“precision”). The O/E-ratios were plotted against their expected values and control limits (95% and 99.8%) were included around the target value (O/E = 1). O/E-ratios of laboratories outside the control limits are considered outliers and perform significantly different from the target value. Additionally, laboratories that lie between both control limits can be considered as random variation.

#### Explaining and understanding laboratory variation in proportion HGD diagnosis

To study whether case-mix at least partly explained the laboratory variation, we compared factor scores based on the standardized proportions with the factors scores of unstandardized proportions. Any explanatory influence of our case-mix variables should result in lower factor scores.

In addition, to further explain the variation, laboratories with low and high standardized HGD proportions were compared for laboratory type, number of reports, previous assessment by another laboratory (i.e., if a different laboratory previously assessed tissue of the same patient) and degree of dysplasia. For this, the three laboratories with the lowest three standardized HGD proportions and the three laboratories with highest three standardized HGD proportions were selected.

#### Sensitivity analysis

When the Spigelman classification was introduced in 1989, the dysplasia grading was originally graded as mild, moderate or severe, which was changed into a two-tiered system (low-grade dysplasia (LGD) and HGD) to decrease interobserver variability [18]. To study the effect of this change on our data, we performed a sensitivity analysis in which only data between 2000 and 2020 were included, after the introduction of the two-tiered system. Laboratory variation and magnitude of variation were calculated as described above.

Analyses were performed with R version 1.3.1073 (R Foundation for Statistical Computing, Vienna, Austria. URL: http://www.R-project.org/) and IBM SPSS Statistics version 25 (SPSS Inc., Chicago, IL, USA).

## Results

### Lab, patient and specimen characteristics

Table 1 shows the laboratory, patient and specimen characteristics. Eight of 25 (32.0%) included laboratories were academic. Mean number of patients and specimens per laboratory was 42 (range 11–127) and 122 (range 27–748), respectively. Mean age of patients at the time of their first report was 55.7 (± 18.3) years, and 43.3% of patients was female. A median of 3 (IQR 5) specimens per patient was included in the analyses. Most specimens (95.5%) were histologically reported from 2001 to 2020. In more than half of reports (53.7%), one specimen was described. Most specimens (84.2%) came from the descending duodenum (D2), with a minority (14.4%) located at the papilla major. HGD was diagnosed in 9.4% of the specimens. Morphology was described as tubular in 63.7%. Only 15.6% of specimens were obtained by polypectomy.

**Table 1:** Laboratory, patient and specimen characteristics

Laboratory characteristics	N = 25
Academic laboratory, n (%)	8 (32.0)
Patients, mean (min–max)	42 (11–217)
Specimens, mean (min–max)	122 (27–748)

Patient characteristics	N = 926
Age (y), mean ± SD	55.7 (18.3)
Sex (female), n (%)	402 (43.3)
Number of specimens, median (IQR)	3 (5)

Specimen characteristics	N = 3050
Year of histology report, n (%)	
1991–2000	137 (4.5)
2001–2020	2913 (95.5)
Removed specimens per report, n (%)^a^	
1	1639 (53.7)
2	698 (22.9)
3	314 (10.3)
4	158 (5.2)
≥ 5	241 (7.9)
Location, n (%)	
D1	232 (7.6)
D2	2569 (84.2)
D3/4	249 (8.2)
Localization at papilla, n (%)	440 (14.4)
Degree of dysplasia, n (%)	
No dysplasia	172 (5.6)
LGD	2580 (84.6)
HGD	287 (9.4)
CA	11 (0.4)
Morphology type, n (%)	
Tubular	1431 (63.7)
Tubulo-villous	717 (31.9)
Villous	100 (4.4)
Removed by polypectomy, n (%)	477 (15.6)

N number; min minimum; max maximum; y years; SD standard deviation; IQR interquartile range; LGD low grade dysplasia; HGD high grade dysplasia; D1 duodenal bulb, D2 descending duodenum, D3 inferior duodenum, D4 ascending duodenum; ^a^ for subsequent analysis only one specimen per report was selected

### Laboratory variation in proportion of HGD diagnosis

**Fig. 2:**
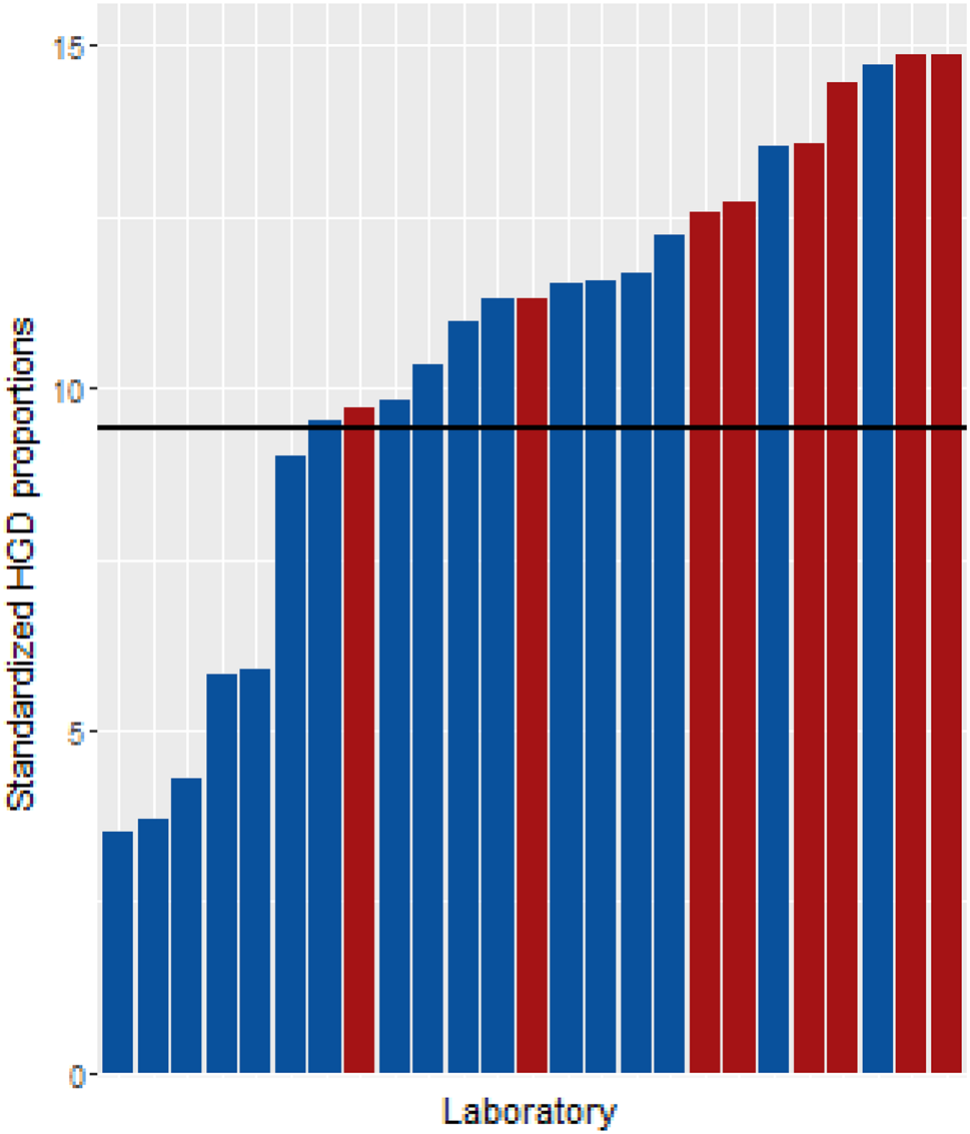
Bar chart representing standardized HGD proportions per laboratory. The horizontal line illustrates the mean observed HGD proportion, which is 9.4%. Red bars indicate academic laboratories. Blue bars indicate general laboratories. HGD high grade dysplasia. (Color figure online).

Figure 2 shows the standardized HGD proportions per laboratory as well as the overall mean. The overall mean observed HGD proportion was 9.4%. The highest standardized HGD proportion was 14.9%, whereas the lowest was 3.5%. All academic laboratories reported more HGD diagnoses than average. The mean highest 3/lowest 3 factor score for the standardized HGD proportions was 3.9, which indicates that tissue diagnosed in the highest 3 diagnosing laboratories had a 3.9 times higher likelihood of being diagnosed as HGD than tissue diagnosed in a laboratory from the lowest 3 laboratories.

**Fig. 3:**
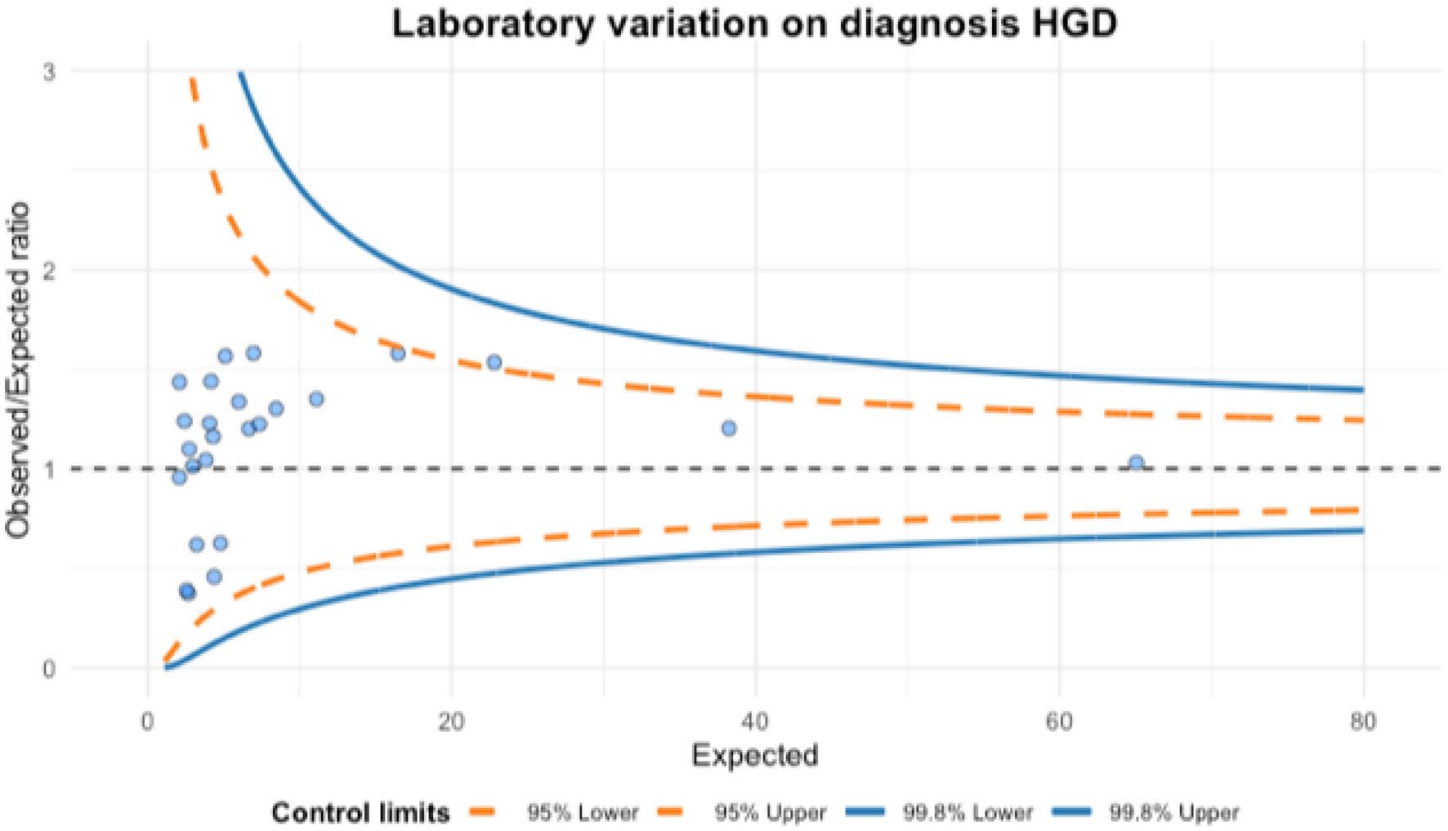
Funnel plot representing the variance between all laboratories

Figure 3 presents a funnel plot showing the variation between laboratories. The O/E (i.e., standardized) ratio is presented on the y-axis, and “expected”, the number of expected HGD-cases per laboratory, on the x-axis. The O/E-ratios varied from 0.4 to 1.6. One laboratory (i.e., 4% of all laboratories) were located outside the 95% control limits. Nonetheless, all laboratories fell within the 99.8% control limits, according to what was expected.

#### Explaining and understanding laboratory variation in proportion of duodenal HGD diagnoses

To investigate the effect of case-mix adjustment on the extent of variation, we compared factor scores based on standardized proportions with the factors scores of unstandardized proportions. The mean factor score for the unstandardized HGD proportions was 7.8, which decreased to 3.9 after case-mix adjustment. The lower factor scores for standardized proportions indicate that our case-mix only partially (3.9/7.8 = 50%) could explain the observed (unstandardized) laboratory variation.

To further identify explanations for variation, Table 2 shows direct comparisons between laboratories with low standardized HGD proportions (lowest 3 laboratories) and laboratories with high standardized HGD proportions (highest 3 laboratories). The lowest 3 laboratories were all general laboratories, while the two of the three highest laboratories were academic laboratories. In the lowest 3 laboratories a mean of 70.7 reports (range 55–91) were included, whereas in the highest 3 laboratories a mean of 101.7 (range 55–165) reports were included. HGD was diagnosed approximately seven times more frequently in the highest 3 laboratories compared to the lowest 3 laboratories (14.0% vs 1.9%, respectively). Both highest and lowest 3 laboratories particularly diagnosed tissue from patients who had not yet been assessed by another laboratory (93.1% vs 94.8%, respectively).

**Table 2:** Characteristics of top 3 and bottom 3 laboratories

	Laboratories with lowest standardized HGD proportion 212 reports (n = 3)	Laboratories with highest standardized HGD proportion 321 reports (n = 3)
Type of laboratory, n (%)		
Academic	0 (0)	2 (66.6)
General	3 (100)	1 (33.3)
Number of patients, mean (min–max)	35.7 (25–42)	38.0 (24–63)
Number of reports, mean (min–max)	70.7 (55–91)	101.7 (55—165)
Previous assessment by different laboratory, n (%)^a^	201 (94.8)	299 (93.1)
No		
Degree of dysplasia, n (%)		
No dysplasia	5 (2.4)	18 (5.6)
LGD	202 (95.3)	256 (79.8)
HGD	4 (1.9)	45 (14.0)
CA	1 (0.5)	2 (0.6)

*Min* minimum, *max* maximum, *LGD* low grade dysplasia, *HGD* high grade dysplasia, *CA* cancer

^a^Indicating if a different laboratory previously assessed tissue of the same patient

### Sensitivity analysis

Sensitivity analysis did not reveal significant differences in highest 3/lowest 3 factor scores (4.25 for standardized proportions), which indicates that the change in dysplasia grading in 2000 did not significantly influence our data. See Supplementary Table 2 for all factor scores.

## Discussion

This study in a Dutch nationwide cohort shows that there is moderate laboratory variation in scoring HGD in duodenal adenomas of FAP patients, as indicated by standardized HGD proportions. Additional explanatory analyses showed that the case-mix of this study could explain approximately half of the observed laboratory variation. The highest three HGD diagnosing laboratories also had higher volumes compared to the lowest three HGD diagnosing laboratories.

### Perspective

Our results showed an observed HGD-proportion of 9.4%. In addition, HGD was diagnosed in 7.0% and 10.7% reports from general versus academic laboratories, respectively. Previous literature on the prevalence of HGD in duodenal polyps in FAP patients is scarce. However, two recent studies briefly discussed this. First, Sourrouille et al. reported that 3.9% of their included cases were diagnosed with HGD after the first upper endoscopy. Five and 10-year rates of HGD were 12.1% and 20.8%, respectively [14]. Second, Roos et al. reported that 4% and 17% of endoscopically removed polyps from the duodenum and papilla were diagnosed as HGD, respectively [22]. Both studies were performed in tertiary centers. Therefore, our results (both overall HGD-proportion as well as the HGD-proportion in academic laboratories) fell within the previously reported range of HGD prevalences.

To our knowledge, this is the first study to report the laboratory variation in HGD scoring of duodenal adenomas in patients with FAP. It is important to realize that the same guideline is currently used for the grading of dysplasia for both colorectal and duodenal tissue [18]. A Dutch study by Kuijpers et al. showed considerable laboratory variability in dysplasia grading of colorectal adenomas, as illustrated by the fact that 13 of 37 (35%) included laboratories were aberrant, i.e. they reported a significantly lower or higher frequency of HGD in colorectal adenomas than average, even after correcting for case-mix. Most of these aberrant laboratories (9/13, 69.2%) reported more HGD than expected, which is in line with our results as standardized proportions of HGD were higher than average in 19/25 (76.0%) laboratories. The effect of volume differences per laboratory was not analyzed in this study [19]. Our results show that the highest 3 laboratories graded more polyps than the lowest 3 laboratories. Yet, as colorectal polyps are approximately four times more common than duodenal polyps [23, 24], it can be expected that the overall volume of graded polyps will make a larger difference in our study, especially as the differences between low- and high-volume laboratories are larger. As an explanation, Kuijpers et al. mention that the subjective criteria for defining dysplasia leave room for variation in interpretation among diagnosing pathologists, which will likely also increase laboratory variation.

### Explaining the variation

In a first attempt to explain the variation we corrected for case-mix, which reduced the variation by approximately 50%, as shown by a reduction of the factor score from 7.8 to 3.9. This indicates that characteristics of the patient populations varied between laboratories. Our data also showed that more reports were included from the three highest HGD diagnosing laboratories compared to the three lowest HGD diagnosing laboratories (101.7 vs. 70.7 reports, respectively). In addition, only 34.1% of the reports were included from 17 general laboratories, whereas 65.9% of the reports were included from 8 academic laboratories. This indicates that small volume (mostly general) laboratories diagnosed HGD less frequently in duodenal tissue from FAP patients than large volume (mostly academic) laboratories.

Differences in HGD proportions between large- and small volume laboratories might be further explained in two ways. First, international guidelines recommend that FAP patients with extensive duodenal or ampullary disease should be referred to high-volume expert centers to consider (endoscopic or surgical) resection [9, 10, 25]. Our descriptive data showed that patients in high-volume laboratories had an older mean age (60 years) than those in low-volume laboratories (55 years, p = 0.04). Since patients in high-volume (referral) centers are older, it seems logical that their duodenal disease was more extensive as it is known that the severity of duodenal polyposis increases with age [26]. In addition, high-volume laboratories reported more specimens per report than low-volume laboratories (1.31 vs 1.15, p = 0.039), probably as a result of more extensive duodenal disease. This may lead to a higher probability of diagnosing HGD. Second, it may also be possible that a pathologist working in a small volume laboratory and therefore less frequently examining duodenal adenomas has more difficulties with diagnosing HGD; however solid evidence for this is as far as we know not available.

Based on current literature and guidelines, there are three other possible explanations to explain the variation in diagnosing HGD in the present study. First, (inter)national guidelines vary regarding the procedure and timing to sample duodenal tissue. For instance, the European Society for Gastrointestinal Endoscopy (ESGE) was the first in 2019 to discourage routine biopsies of suspected lesions in the duodenum for FAP patients, as this may cause fibrosis which may lead to difficulties in future possible endoscopic resection [9]. But even before 2019 this was already done in some hospitals [27]. In addition, conflicting recommendations exist regarding (endoscopic or surgical) resection of duodenal tissue. For example, the ESGE recommends polyp size ≥ 10 mm as indication for endoscopic resection, while the Netherlands Foundation for Detection of Hereditary Tumors recommends resection when Spigelman stage IV disease, HGD or growing papillary adenomas are found [9, 28]. This varying recommendations between guidelines will probably lead to variation in tissue sampling between hospitals. In turn, this might have led to differences in the quantity of duodenal tissue to be graded and hence the probability of diagnosing HGD per laboratory.

Second, criteria to grade dysplasia are subjective and depending on the interpretation by pathologists. Kuijpers et al. performed a questionnaire study that showed considerable heterogeneity in the criteria applied by pathologists to grade dysplasia within colorectal adenomas [19]. As a possible consequence, several studies evaluating interobserver variability in dysplasia grading of colorectal adenomas have shown widely varying results from poor to good agreement between pathologists (κ = 0.02–0.69) [29–35]. Subjectivity within a diagnosing guideline can lead to both under- and overdiagnosis of HGD as pathologists might suffer from ‘professional uncertainty’. The latter is hypothesized to occur when physicians are uncertain about a clinical decision [36, 37]. For pathologists this means that heterogeneity in diagnostic criteria for HGD may lead to insecurity in diagnosing it, which in turn may lead to variation in HGD diagnosis.

Third, over the years (inter)national and even local protocols have been inconsistent regarding the clinical consequences of a HGD diagnosis for duodenal tissue. The American Society for Gastrointestinal Endoscopy (ASGE) guideline recommends considering endoscopic therapy for a lesion with HGD, whereas the ESGE guideline does not give a clinical recommendation when HGD is diagnosed, even though HGD is regarded as a risk factor for developing duodenal cancer [9, 11]. Moreover, long-term data on the effect of endoscopic resection of duodenal polyposis in FAP patients is only limited available [27]. Though we were unable to collect information on the local protocols for management of a HGD diagnosis per hospital, it is likely that these differed between hospitals. It may well be that this variation in local protocols (further) causes professional uncertainty as pathologists are uncertain about the subsequent consequences after HGD is diagnosed, leading to both over- and underdiagnosis of HGD.

### Future prospects

Various developments have been implemented to improve the diagnosis of HGD in pathology laboratories. Recently, five FAP expertise centers in the Netherlands were selected to clinically manage the disease, including the histological diagnosis of HGD. It can be expected that this will decrease the interlaboratory variation and misdiagnoses of HGD and increase uniformity in HGD grading. Due to centralization, only dedicated gastroenterologists and pathologists will be involved in the diagnostic process, which is expected to reduce interobserver variability [29, 38]. Moreover, as suggested in previous studies, multidisciplinary team meetings may further reduce interobserver variability [32, 39, 40]. Future research should show if laboratory variation has indeed reduced when histopathological diagnosis in mainly performed in expert centers.

It is clear that too much subjectivity in diagnosing HGD is unwarranted. We therefore encourage better standardization of histologic grading criteria for duodenal adenomas. In addition, previous literature has shown that the implementation of an e-learning improves interobserver variability in Dutch laboratories regarding the grading of colorectal dysplasia [41]. There is no reason to believe that widespread implementation of e-learnings also will decrease variation in grading of duodenal dysplasia. Furthermore, the use of artificial intelligence has the potential to decrease variation in HGD diagnosis. However, current research is limited to the recognition of colorectal dysplasia (without subdividing it into low- or high grade) and carcinomas [42, 43]. Therefore, future research is warranted to investigate the role of artificial intelligence in diagnosing HGD in duodenal adenomas.

It is also important to make current guidelines on polyposis syndromes more consistent regarding taking biopsies from duodenal polyps, and to define a uniform clinical strategy when HGD in duodenal polyps is diagnosed (i.e., HGD as an indication for endoscopic or surgical interventions or not). The clinical guidelines from the European Hereditary Tumour Group (EHTG) on polyposis syndromes are currently being revised. This gives the opportunity for at least European guidelines to become more uniform in their recommendations.

### Strengths and limitations

Our study has several strengths and limitations. A definite strength is that we were able to use nationwide, longitudinal data, including a cohort of 1217 patients (5782 pathology reports), which is large as FAP is a rare disorder. Second, we were able to show laboratory variation in clinical practice, rather than in a controlled study design, as was the case in previous interobserver variability studies [29–35].

In addition, some limitations should be addressed as well. First, inherent to the data source, specified clinical data on patient characteristics (e.g., age at first upper endoscopy and genetic mutation), endoscopic findings (e.g., size of polyps observed during upper endoscopy) and laboratory specifications (e.g., practices of double reading) were not available. These characteristics are all known to be predictive factors for developing HGD and duodenal cancer [13, 14]. Nevertheless, it is known that endoscopic characteristics of duodenal polyposis in FAP patients are poorly reported, as the multiplicity of the polyps impedes exact documentation of number, size and location of the polyps. It is unknown whether this additional information from endoscopy reports would have changed our findings. Second, while we created a large cohort of 1217 patients, the data were collected over a period of 29 years. However, our sensitivity analysis did not show any remarkable differences, which indicates that our results are fairly robust.

## Conclusion

Laboratory variation in histological grading of duodenal adenomas of FAP patients was found to be moderate. Patient characteristics considerably explained the variation, indicating patient populations differed between hospitals. Still, there is considerable variation, which leaves room for quality improvement. We are optimistic that the nationwide laboratory variation will decrease with the centralization of care for patients with FAP in five expertise centers in the Netherlands. However, further standardization of the grading criteria for dysplasia of gastro-intestinal and thus duodenal adenomas is needed and (inter)national guidelines should become more uniform regarding the necessity to routinely take biopsies from duodenal polyps and on the clinical consequences of a HGD diagnosis in FAP patients to decrease unwarranted laboratory variation.

## Supplementary Information

Below is the link to the electronic supplementary material.

Supplementary file1 (DOCX 13 kb)

Supplementary file2 (DOCX 13 kb)

## Data Availability

The datasets generated during and/or analysed during the current study are available from the corresponding author on reasonable request.

## References

[1] Jasperson KW, Tuohy TM, Neklason DW (2010). Hereditary and familial colon cancer. Gastroenterology.

[2] Kanth P, Grimmett J, Champine M (2017). Hereditary colorectal polyposis and cancer syndromes: a primer on diagnosis and management. Am J Gastroenterol.

[3] Bodmer WF, Bailey CJ, Bodmer J (1987). Localization of the gene for familial adenomatous polyposis on chromosome 5. Nature.

[4] Powell SM, Petersen GM, Krush AJ (1993). Molecular diagnosis of familial adenomatous polyposis. N Engl J Med.

[5] Karstensen JG, Burisch J, Pommergaard HC (2019). Colorectal cancer in individuals with familial adenomatous polyposis, based on analysis of the Danish polyposis registry. Clin Gastroenterol Hepatol.

[6] Belchetz LA, Berk T, Bapat BV (1996). Changing causes of mortality in patients with familial adenomatous polyposis. Dis Colon Rectum.

[7] Offerhaus GJ, Giardiello FM, Krush AJ (1992). The risk of upper gastrointestinal cancer in familial adenomatous polyposis. Gastroenterology.

[8] Syngal S, Brand RE, Church JM, et al (2015) ACG clinical guideline: Genetic testing and management of hereditary gastrointestinal cancer syndromes. Am J Gastroenterol 110:223–262;quiz 263. 10.1038/ajg.2014.435 PMC4695986 25645574

[9] van Leerdam ME, Roos VH, van Hooft JE (2019). Endoscopic management of polyposis syndromes: European Society of Gastrointestinal Endoscopy (ESGE) Guideline. Endoscopy.

[10] Monahan KJ, Bradshaw N, Dolwani S (2020). Guidelines for the management of hereditary colorectal cancer from the British Society of Gastroenterology (BSG)/Association of Coloproctology of Great Britain and Ireland (ACPGBI)/United Kingdom Cancer Genetics Group (UKCGG). Gut.

[11] Yang J, Gurudu SR, Koptiuch C, et al (2020) American Society for Gastrointestinal Endoscopy guideline on the role of endoscopy in familial adenomatous polyposis syndromes. Gastrointest Endosc 10.1016/j.gie.2020.01.028 32169282

[12] Spigelman AD, Williams CB, Talbot IC (1989). Upper gastrointestinal cancer in patients with familial adenomatous polyposis. Lancet.

[13] Thiruvengadam SS, Lopez R, O'Malley M (2019). Spigelman stage IV duodenal polyposis does not precede most duodenal cancer cases in patients with familial adenomatous polyposis. Gastrointest Endosc.

[14] Sourrouille I, Lefevre JH, Shields C (2017). Surveillance of duodenal polyposis in familial adenomatous polyposis: should the Spigelman score be modified?. Dis Colon Rectum.

[15] Roos VH, Bastiaansen BAJ, Dekker E (2019). Challenges and pitfalls of investigating duodenal cancer in patients with familial adenomatous polyposis. Gastrointest Endosc.

[16] Westert GP, Groenewoud S, Wennberg JE (2018). Medical practice variation: public reporting a first necessary step to spark change. Int J Qual Health Care.

[17] Wennberg JE (2002). Unwarranted variations in healthcare delivery: implications for academic medical centres. BMJ.

[18] Schlemper RJ, Riddell RH, Kato Y (2000). The Vienna classification of gastrointestinal epithelial neoplasia. Gut.

[19] Kuijpers CC, Sluijter CE, von der Thusen JH (2016). Interlaboratory variability in the grading of dysplasia in a nationwide cohort of colorectal adenomas. Histopathology.

[20] Casparie M, Tiebosch AT, Burger G (2007). Pathology databanking and biobanking in The Netherlands, a central role for PALGA, the nationwide histopathology and cytopathology data network and archive. Cell Oncol.

[21] Wennberg JE (2011). Time to tackle unwarranted variations in practice. BMJ.

[22] Roos VH, Bastiaansen BA, Kallenberg FGJ, et al (2020) Endoscopic management of duodenal adenomas in patients with familial adenomatous polyposis. Gastrointest Endosc 10.1016/j.gie.2020.05.065 32535190

[23] Jepsen JM, Persson M, Jakobsen NO (1994). Prospective study of prevalence and endoscopic and histopathologic characteristics of duodenal polyps in patients submitted to upper endoscopy. Scand J Gastroenterol.

[24] Pan J, Cen L, Xu L (2020). Prevalence and risk factors for colorectal polyps in a Chinese population: a retrospective study. Sci Rep.

[25] Yang JG, Koptiuch C, Agrawal D, Buxbaum JL, Abbas Fehmi SM, Fishman DS, Khashab MA, Jamil LH, Jue TL, Law JK, Lee JK, Naveed M, Qumseya BJ, Sawhney MS, Thosani N, Wani SB, Samadder NJ (2020) American Society for Gastrointestinal Endoscopy guideline on the role of endoscopy in familial adenomatous polyposis syndromes. Gastrointest Endosc 10.1016/j.gie.2020.01.028 32169282

[26] Bulow S, Bjork J, Christensen IJ (2004). Duodenal adenomatosis in familial adenomatous polyposis. Gut.

[27] Soons E, Bisseling TM, van Kouwen MCA (2021). Endoscopic management of duodenal adenomatosis in familial adenomatous polyposis—a case-based review. United Eur Gastroenterol J.

[28] Tumors TNFfDoH (2017) Erfelijke Familiaire Tumoren - Richtlijnen voor diagnostiek en preventie.

[29] Terry MB, Neugut AI, Bostick RM (2002). Reliability in the classification of advanced colorectal adenomas. Cancer Epidemiol Biomark Prevent.

[30] van Putten PG, Hol L, van Dekken H (2011). Inter-observer variation in the histological diagnosis of polyps in colorectal cancer screening. Histopathology.

[31] Foss F, Milkins S, McGregor A (2012). Inter-observer variability in the histological assessment of colorectal polyps detected through the NHS Bowel Cancer Screening Programme. Histopathology.

[32] Turner JK, Williams GT, Morgan M (2013). Interobserver agreement in the reporting of colorectal polyp pathology among bowel cancer screening pathologists in Wales. Histopathology.

[33] Mahajan D, Downs-Kelly E, Liu X (2013). Reproducibility of the villous component and high-grade dysplasia in colorectal adenomas <1 cm: implications for endoscopic surveillance. Am J Surg Pathol.

[34] Lasisi F, Mouchli A, Riddell R (2013). Agreement in interpreting villous elements and dysplasia in adenomas less than one centimetre in size. Dig Liver Dis.

[35] Osmond A, Li-Chang H, Kirsch R (2014). Interobserver variability in assessing dysplasia and architecture in colorectal adenomas: a multicentre Canadian study. J Clin Pathol.

[36] Birkmeyer JD, Reames BN, McCulloch P (2013). Understanding of regional variation in the use of surgery. Lancet.

[37] Wennberg J, Gittelsohn (1973) Small area variations in health care delivery. Science 182:1102–1108. 10.1126/science.182.4117.1102 4750608

[38] Denis B, Peters C, Chapelain C (2009). Diagnostic accuracy of community pathologists in the interpretation of colorectal polyps. Eur J Gastroenterol Hepatol.

[39] Smits LJH, Vink-Börger E, van Lijnschoten G, et al (2021) Diagnostic variability in the histopathological assessment of advanced colorectal adenomas and early colorectal cancer in a screening population. Histopathology 10.1111/his.14601 PMC9306715 34813117

[40] Rampioni Vinciguerra GL, Antonelli G, Citron F (2019). Pathologist second opinion significantly alters clinical management of pT1 endoscopically resected colorectal cancer. Virchows Arch.

[41] Madani A, Kuijpers C, Sluijter CE (2019). Decrease of variation in the grading of dysplasia in colorectal adenomas with a national e-learning module. Histopathology.

[42] Wang KS, Yu G, Xu C (2021). Accurate diagnosis of colorectal cancer based on histopathology images using artificial intelligence. BMC Med.

[43] Ho C, Zhao Z, Chen XF (2022). A promising deep learning-assistive algorithm for histopathological screening of colorectal cancer. Sci Rep.

