# Supplementary material for: Laboratory variation in the grading of dysplasia of duodenal adenomas in familial adenomatous polyposis patients
Source: Fam Cancer. 2022 Nov 19;22(2):177–86. doi: 10.1007/s10689-022-00320-1 (PMC10020317; doi:10.1007/s10689-022-00320-1)
Supplement: Supplementary file 1 — Supplementary file1 (DOCX 13 kb) [file 10689_2022_320_MOESM1_ESM.docx]

| **Patient characteristic** | **Odds-ratio (95%-CI)** | **P-value** |
| --- | --- | --- |
| Age | 1.03 (1.02 – 1.05) | <0.001* |
| Year of report (2001 – 2020) | 0.38 (0.18 – 0.82) | 0.01* |
| Number of specimens per report | 1.12 (1.04 – 1.22) | 0.005* |
| Localization in the duodenum  D2  D3/4 | 1.21 (0.61 – 2.40)  0.71 (0.28 – 1.83) | 0.58  0.48 |
| Localization at papilla major | 1.15 (0.70 – 1.90) | 0.58 |
| Morphology  Tubulo-villous  Villous | 4.34 (2.96 – 6.35)  4.33 (2.16 – 8.68) | <0.001*  <0.001* |
| Removed by polypectomy | 1.35 (0.87 – 2.08) | 0.18 |

**Supplementary Table 1** Contribution per case-mix variable on logistic regression model with laboratory level. CI confidence interval; D2 descending duodenum; D3/4 inferior/ascending duodenum; * significant Odds-ratio.
