# Supplementary material for: Laboratory variation in the grading of dysplasia of duodenal adenomas in familial adenomatous polyposis patients
Source: Fam Cancer. 2022 Nov 19;22(2):177–86. doi: 10.1007/s10689-022-00320-1 (PMC10020317; doi:10.1007/s10689-022-00320-1)
Supplement: Supplementary file 2 — Supplementary file2 (DOCX 13 kb) [file 10689_2022_320_MOESM2_ESM.docx]

|  | **1991 - 2020** | | **2000 - 2020** | |
| --- | --- | --- | --- | --- |
|  | **Observed** | **Standardized** | **Observed** | **Standardized** |
| Top 3 / Bottom 3 | 7.76 | 3.86 | 7.70 | 4.25 |

**Supplementary Table 2** Factor scores for main analysis (1991 – 2020) versus sensitivity analysis (2000 – 2020).
